# Family violence against children in the wake of COVID-19 pandemic: a review of current perspectives and risk factors

**DOI:** 10.1186/s13034-020-00347-1

**Published:** 2020-10-20

**Authors:** Noemí Pereda, Diego A. Díaz-Faes

**Affiliations:** Grup de Recerca en Victimització Infantil i Adolescent (GReVIA), Facultat de Psicologia,, Universitat de Barcelona, Passeig Vall d’Hebron, 171, 08035 Barcelona, Spain

**Keywords:** COVID-19, Violence, Developmental victimology, Risk factors, Children

## Abstract

The situation of crisis produced by the Coronavirus (COVID-19) pandemic poses major challenges to societies all over the world. While efforts to contain the virus are vital to protect global health, these same efforts are exposing children and adolescents to an increased risk of family violence. Various criminological theories explain the causes of this new danger. The social isolation required by the measures taken in the different countries, the impact on jobs, the economic instability, high levels of tension and fear of the virus, and new forms of relationships have all increased levels of stress in the most vulnerable families and, therefore, the risk of violence. In addition, mandatory lockdowns imposed to curb the spread of the disease have trapped children in their homes, isolating them from the people and the resources that could help them. In general, the restrictive measures imposed in many countries have not been accompanied by an analysis of the access to the resources needed to reduce this risk. It is necessary to take urgent measures to intervene in these high-risk contexts so that children and adolescents can develop and prosper in a society which is likely to undergo profound changes, but in which the defense of their rights and protection must remain a major priority.

## Background

In terms of their frequency and impact, violence and exposure to violence in the immediate family context are among the most serious forms of victimization [1]. These forms of victimization usually may include physical abuse, psychological or emotional abuse, sexual abuse, neglect, and exposure to intimate partner violence [2]. The worldwide estimations of prevalence in self-report surveys are 22.6% for physical abuse, 36.3% for emotional abuse, 7.6% among boys and 18% among girls for sexual abuse, 16.3% for physical neglect, and 18.4% for emotional neglect [3]. Regarding childhood exposure to intimate partner violence, the data available are still limited [4]; however, it is estimated that between 133 and 275 million children are exposed to this kind of violence each year [5]. In spite of the gaps in the measurement of child victimization [6], these high rates of violence and exposure to family violence reveal that interpersonal violence against children constitutes a serious global social and health problem [7].

As the COVID19 crisis continues, societies all over the world are trying to mitigate its effects in both the short and the long term. Besides looking at the risks and consequences for the mental health of children and adolescents arising from the pandemic and the measures imposed to control it [8], a criminological approach, in which the restrictive emergency measures adopted to slow the spread of the virus take into account the risk of family violence, is also essential. Social distancing, school and business closures, lockdown measures, and travel restrictions may reduce the transmission of the infectious disease, but they may also increase the risk of violence against children and youth around the world [9].

For violence researchers, the measures taken in response to COVID-19 present an extraordinary opportunity to advance our understanding of the social, psychological, economic and situational mechanisms that influence rates of violence [10]. In fact, contexts associated with pandemics create an environment in which children’s socioecological systems are disrupted and, as a result, the incidence of child maltreatment is likely to increase [11]. However, many child welfare organizations around the world are noting a significant drop in reports of child abuse or neglect [12]. We know little about the amount of violence that children and adolescents have faced in their homes since the beginning of the pandemic, and institutions like UNICEF [13] have discouraged the performance of epidemiological studies in children unless their protection can be guaranteed. Thus, it is necessary to intervene indirectly by detecting contexts of risk and by exploring the factors that may increase violence against children and youth so as to be able to take successful action.

Therefore, the main objective of this narrative review is to describe the risk factors for violence against children that the situational context derived from COVID-19 may have generated in families around the world, and to assess them from a criminological perspective. In order to gather the latest evidence, we conducted a search of several databases that identify published and preprint research (i.e., PubMed, PsycINFO, Web of Science, Scopus, SSRN, and Google Scholar). The search terms were focused on family violence against children, risk factors and theoretical models, and crisis contexts and/or COVID-19. Here we review the theoretical and empirical studies published to date on the topic of child maltreatment and COVID-19. We also include some references on previous crises and disasters to assess the relevance of those results to the current situation. Finally, we try to explain the more than likely high risk of child abuse during the COVID-19 crisis in the light of criminological, psychological and sociological theories.

## Increasing risk of violence against children during the COVID-19 pandemic

The COVID-19 pandemic may have entailed major changes for many children and their families, not just because of the lockdown, restricted measures, social isolation, changing demographics and the reduction of available health services [14], but also due to the sudden and possibly long-term increase in child poverty and family uncertainty [15]. The pandemic represents a global crisis not only for our health and economy, but also for family well-being through a cascading process of factors that can drive, precipitate or exacerbate potential stressors [16]. The situation generated by COVID-19 has few precedents, but we can build on the work in crisis or emergency situations where scenarios of rapidly increasing stress are accompanied by abrupt changes to prior conditions (see the review by [17]).

The effects of disasters and mass violence on individual development can be described in relation to the exposure dose or cumulative risks that pose significant threats or disturbances to individuals, families, or communities [18]. Thus, the COVID-19 pandemic has been conceptualized as a multisystem cascading global disaster in which children’s lives have been dramatically disrupted at many levels and for which our societies were unprepared [19]. Indeed, research on COVID-19 is beginning to show the negative outcomes of the lockdown and the restrictions imposed as well as the effects of social stressors on family members, and highlights the need for longitudinal examination of children’s and adolescents’ mental health [20]. Emerging evidence on both healthy parenting and the mental health of children and adolescents stresses that the magnitude of the impact depends on vulnerability factors such as developmental age, previous mental health conditions, educational and socioeconomic status, or being quarantined [21].

This dramatically changing context also needs to be understood in order to address the risk of violence against children and adolescents, which is essential if our aim is to prevent or detect these cases before the consequences of this violence are irremediable. In this paper we analyze the risk factors for violence against this population from the perspective of criminological theories and socio-ecological models.

### Increased risk of violence through the lens of criminological theories

Criminological theories address the multiple variables that contribute to family violence and child abuse, and can also explain why there is a greater risk of violence in critical situations. The intergenerational transmission of violence summed up in the term ‘violence begets violence’, is one of the prevalent assumptions in the literature: i.e., that the experience of childhood violence and/or neglect increases the risk of perpetrating violence in later life [22]. Due to the complexity of the concept, our understanding of intergenerational transmission of violence is still limited [23]. Several theories have endeavored to explain the mechanisms involved, such as social learning theories [24], social information processing theory [25] attachment theory [26], and social control theory [27].

In addition, neurobiological studies have revealed the impact of chronic stress due to an abusive childhood, which may cause a neurobiological deregulation that affects neurological and brain development and modifies the response to stress related to violent behavior [28]. Evolutionary behavioral genetics explains the probability that violent parents beget violent children through various mechanisms such as trait inheritance or epigenetic changes predisposing to violent behavior [29].

However, an excessive focus on experiences of child abuse may turn out to be a static or limited, or may even interpret the later consequences as a direct product of the early exposures. Equally, the influence of genetics on violent behavior may be moderated by environmental conditions. Therefore, nature and nurture should be considered not as distinct and separate factors but as part of the same process in human behavior and, in this case, in the violence perpetrated against children in times of crisis [30]. Thus, for a more comprehensive analysis, ecological and contextual factors need to be addressed.

Environmental and situational theories help to understand children’s vulnerability and the contexts that are most conducive to violence against them. Social disorganization theory proposes that neighborhood characteristics such as poverty, residential mobility, population density, overcrowding or urban blight hinder or prevent community cohesion, which lead to the increased levels of disadvantage and disorder associated with high rates of child abuse [31]. Theories of situational opportunity [32] help to understand the change in prior conditions, attributing the greater likelihood of violence to perpetrators’ increased access to victims due to confinement and limitations on mobility.

Two approaches that combine and integrate some elements of the above frameworks or address the same correlates, though from different angles, are the general strain theory, which applies a sociological perspective, and target congruence theory, which applies a developmental victimology approach. General strain theory can contribute to explaining family violence and child abuse from both a proximal and a distal perspective, since it focuses on the impact of negative emotions such as anger, frustration, and resentment on the subsequent violent behavior [33]. The effect may be distal if it derives from childhood or long-term processes (e.g., adverse childhood experiences), or proximal if it derives from sudden events or situations, or it may be a confluence of both. Applied to the COVID-19 pandemic, the general strain theory posits that the interaction of the family and parents’ or caregivers’ background with the increase in stress and intra-family tension caused by the accumulation of risk factors linked to the emergency (and the measures imposed to control it) produces an increase in violence.

Finkelhor and Asdigian’s [34] target congruence theory presents three explanatory factors for the increase in violence against children. Thus, (a) the vulnerability of potential victims, due to characteristics of the context or of the victims themselves that increase the likelihood of victimization, such as their dependence on an adult, their physical weakness, and their greater social isolation; (b) the satisfaction or gratificability generated by the use of violence, whether sexual in the case of sexual abuse and assaults, or as a way of discharging tension in the use of physical and emotional violence; and (c) antagonism, linked to characteristics or attributes of the child that arouse impulses of rejection or violence in the victimizer, such as constant requests for attention and care.

Indeed, the factors and mechanisms displayed converge and interact with the various social stressors [35] triggered or exacerbated by the COVID-19 pandemic. Their study will help to establish whether the body of knowledge accumulated by criminological theories is sufficient to identify some of the pathways that lead towards the perpetration of violence against the young, and also whether the response to the pandemic situation may favor the development of more comprehensive approaches to victimization in this age group.

### Increased risk of violence through the lens of socioecological models

Socioecological accounts can provide a general framework of how the COVID-19 pandemic has directly or indirectly disrupted social ecologies and modified the interaction between individuals and their environment. The changes in this reciprocal relationship may provide new definitions for individuals’ cognitions, emotions, behaviors and the underlying associated mechanisms. So, this mutual process manifests itself in changing physical, interpersonal, economic and political environments and in the ways in which humans adapt and modify these environments [36]. Given that child maltreatment is an interactional phenomenon, the COVID-19 crisis has altered children’s ecological systems at a variety of levels. It has generated or aggravated a series of risk factors for child abuse and neglect pertaining to the characteristics of the child and caregivers, family dynamics, and the wider social and cultural environment. For this reason, we assess these risks through the lens of Belsky’s [37] ecological integration model at its various levels. Within this model, we also consider the transactional process [38] which entails that at each ecological level the complex interaction between potentiating and compensatory factors influences both children themselves and their ecological systems. Ecological levels of analysis capture and systematize the multiple variables contributing to child abuse, but also allow the incorporation of complementary approaches such as sociocognitive approaches to parenting that can help to understand how environmental changes influence the incidence of child abuse [39].

At the level of the microsystem, increased oppositional behavior and limit testing are expected in children. This conduct may elicit harsh responses from parents [40], who may themselves be undergoing parental burnout, either driven or exacerbated by the consequences of the pandemic [41]. Children’s own stress and uncertainty about the pandemic may add to the tension felt by their parents. The COVID-19 pandemic may also worsen existing mental health problems and trigger more cases among children and adolescents [42], thus increasing tension within the home. A recent study found that since the start of the pandemic more than 1 in 4 parents reported worsening mental health in their children, 1 in 7 parents reported worsening behavioral health, and nearly 1 in 10 reported worsening in both [43]. Children with special education needs are also at risk; they may become frustrated and short-tempered due to the disruption of their daily routines [44]. Overall, stressed parents are more likely to respond to their children’s anxious behaviors or demands in aggressive or abusive ways. Initial research has shown that the situation caused by the COVID-19 crisis is highly demanding and challenging for parents and significantly increases their global levels of stress [45]. Previous research has also confirmed that a high-stress home environment is often a major predictor of physical abuse and neglect of children [46].

Parental alcohol consumption at home (as a result of the closure of pubs, bars and hotels) in order to manage stress and tension can also contribute to increasing violence against children [47], as can the presence of pre-existing mental disorders in parents and the lack of monitoring or medication [48]. Parents are under stress and the COVID-19 is changing family life [49]: children are out of school or childcare, and do not have access to group activities, team sports, or playgrounds. Parents have to keep them busy and safe while at the same time attempting to work at home; indeed, they may be unable to work due to their child care duties. Evidence of common correlates suggests that conflicts and violence between parents probably target children as well [50]. The higher increased risk of violence between the parents during quarantine due to COVID-19 might has made it particularly difficult to meet the children’s needs [51].

At the level of the exosystem, or society at large, the economic consequences of the crisis are also risk factors [52]. Lost income, cumulative material adversity and housing hardships are the main predictors of child maltreatment [53]. For those living in low-income and crowded households without open spaces, challenges posed by COVID-19 are exacerbated [49]. Social isolation, in turn, prevents the detection of situations of abuse by people outside the family and stops the parents from seeing other models of relationships with children not based on violence, limiting accessible and familiar support options [54]. While the dramatic decline in social interactions is likely to have limited the contact of children with a wide range of potential reporters of child maltreatment including pediatricians and extended family members, the forced closure of nurseries and schools may also have significantly affected both the reporting and the incidence of cases of child maltreatment; this is especially worrying in view of the low levels of reporting in normal conditions [55]. Public nurseries represent an important protective factor against maltreatment [56] but their ability to play this role has been curtailed by COVID-19. Also, school closures have meant an estimated 27% decline in the reported allegations of abuse, neglect, or abandonment of children made to the Florida Child Abuse Hotline during March and April 2020, a reduction very similar to that seen in normal times when school is out of session [57]. In addition, the resources that many at-risk parents rely on are no longer available in many areas in times of crisis. Recent research has highlighted the restrictions experienced by the NGOs around the world in their attempts to provide services to vulnerable children and families during confinement [58].

Finally, with regard to culture, attitudes towards children and their rights constitute a key risk factor for violence in the COVID-19 context. Even though research has shown that children are not the main drivers of the pandemic [59], in many countries they have been accused of being vectors of transmission of the virus, even by official leaders, and are considered more contagious than asymptomatic adults. This may have led to a degree of social rejection and may have engendered a lack of empathy for the serious consequences of home-confinement for their development and well-being [60]. In fact, even though adult society is returning to normal, schools, kindergartens or even playgrounds in most countries remain closed [61]. The treatment of this crisis is absolutely adult-centered—that is, focused on the needs and criteria of adults—and has neglected the needs of the most vulnerable and their protection. The UN Convention on the Rights of the Child is being ignored in most countries, both during the lockdown and since [62]. As a result, the increase in violence against children both during and since the pandemic may even surpass the substantial increase characteristically observed in reports following natural disasters and other catastrophic events.

In view of the above, some of the core principles of the risk-need-responsivity model [63] could be applied to the prevention of child victimization [64]. Knowledge of the risk factors is vital for preventing or reducing violence, and for identifying the most suitable forms of support and intervention in order to address these risks in a proportionate manner. This is a crucial aspect considering that methods of prevention will need to adapt to the post-COVID-19 scenario.

## Conclusion

The present review is one of the few studies to date that has focused on the risk factors for family violence against children and youth related to the COVID-19 pandemic. We have sought to explain this risk through the application of criminological theories and socioecological models, stressing the importance of the social sciences when dealing with a pandemic and its massive global health consequences [65]. We can conclude that, in the unprecedented situation produced by the COVID-19 crisis, the risk of victimization of children and adolescents is high. The repercussions of the COVID-19 pandemic go far beyond the measures to prevent disease transmission and reduce its impact on the global population [66]. Child victim services and family violence victim-serving professionals must be prepared for the likely increases in victimization rates both during and long after this pandemic. They must address the new obstacles and look for innovative solutions based on a community-centered approach [67]. It is also vital to identify high-risk contexts in order to avoid the occurrence of long-term new acts of violence [68]. Although an initial rise in family violence is generally observed during the acute phase of a crisis, the fact we need to keep in mind is that these surges are often sustained for years during the recovery period and require a prevention strategy that offers long-term solutions [69]. The potential consequences of increased child maltreatment should be considered in future cost–benefit calculations of lockdown measures. Since family violence must be seen as a possible public health consequence of the COVID-19 pandemic, governments should work together with social care and health care providers to integrate child maltreatment into future plans for disaster risk reduction and preparedness [70]. It is crucial to learn as much as possible from this disaster to prepare for similar (or totally unprecedented) crisis situations in the future [19]. We should take the necessary measures to enable children and adolescents to develop and prosper in a society which is likely to be very different, but in which the defense of their rights and well-being must continue to be a priority.

## Data Availability

Not applicable.
